# Diphtheria—Croup—O’Dwyer’s Method—Recovery

**Published:** 1886-05-20

**Authors:** 


### DIPHTHERIA—CROUP—O'DWYER'S METHOD—RECOVERY.

The following is the report of a case occurring in the practice of Dr. R. N. Disbrow, of this city:

The patient, a child two years and nine months old, was seen by the doctor for the first time on March 8, 1886. The pharynx was covered with diphtheritic exudate; the cough was slightly croupy, and the general condition was poor. At that time she had been ill five days; "started to lay around and got feverish."

March 9th.—Croup well marked; there are croupy cough, croupy inspiration and expiration, and she had been restless and sleepless all night. At 2 p. m., I saw the case with Dr. Disbrow. The child's complexion was of a dull, pale color, but respiration was loud and hoarse, the cough hard and croupy, and her voice entirely gone. She was very restless, turning over and over on the bed, making it impossible to keep her covered, throwing her arms, and tearing at the clothes about her neck. There was marked recession of the chest-walls on inspiration. On auscultation, no vesicular breathing could be heard over the back. On examining the throat, the slightest embarrassment to breathing brought on deep cyanosis. The entire pharynx was covered with diphtheritic exudate; there were also patches on the tongue and lips. Besides internal remedies, steam had been thoroughly tried, and at the time slaking lime was doing its best.

The doctor advised surgical interference, but the parents positively declined. They had known of a case of tracheotomy, and would not consent. He then represented to them the gravity of the case, and repeatedlv assured them there would be "no ether, no cutting, no blood. They consented, and at 4 p.m., in the presence of Dr. Disbrow, Dr. O'Dwyer, and Dr. Booth, I inserted a tube estimated to be of the proper size for a three year old child.

The usual method of inserting the tube is as follows: Wrap the child in a blanket, set it up straight on a nurse's lap squarely facing the operator, and insert the mouth-gag, having a third person to steady the head. With the left index-finger hook up the epiglottis and with the right hand insert the tube bv means of a holder, and detach it. When assured that it is in the larynx and not in the œsophagus, withdraw the thread and wait.

In this case the insertion required about five seconds. The child looked bewildered, began to swallow, loose mucus rattled and provoked moderate cough, and the child's color changed from dusky to clear. In ten minutes she was sleeping in bed so quietly that her respiration was inaudible at a distance of two feet. Having relieved the laryngeal obstruction, we all went home, leaving an unpromising case in the hands of an old man as nurse. At 8 p.m., the child was given some milk, which she swallowed with difficulty—it caused her to cough. She had slept most of the time since 4 o'clock. Temperature 100.5 F., pulse 144, respiration 18.

10th.—She sleeps well, coughs little, and swallows with some difficulty two pints of milk and some beef-tea. Temperature in the rectum 100 F., pulse 144, respiration 36. There is good vesicular breathing over both lungs.

15th.—Vesicular breathing good; pharynx clear of all exudate; general condition good; respiration easy. She coughs more, but swallows much better. The child smiles and sits up in bed and plays with her toys.

16th.—We had planned to remove the tube, when on our visit it was found that the patient had coughed it out at 4 a.m., just six days and a half from its insertion. Temperature in the rectum 99.5 F., pulse 112, respiration 24, and the general condition good. The nurse said that she swallows better since the tube came away, though she had leared to swallow very well with it in. There is complete aphonia.

26th.—The voice begins to return.

29th.—The child talks and sings—thirteen days after the removal of the tube. She runs about, eats well, and has no paralysis.

During the recovery of this patient, a baby, eighteen months old, in the same family, sickened with diphtheria (naso-pharyngeal) and died).—*N. Y. Medical Journal.*

